# Gender mainstreaming in sweetpotato breeding and dissemination in Ghana and Malawi

**DOI:** 10.3389/fsoc.2024.1263438

**Published:** 2024-04-30

**Authors:** Obaiya G. Utoblo, Putri Ernawati Abidin, Eric Kuuna Dery, John K. Bidzakin, Netsayi N. Mudege, Isaac Korku Dorgbetor, Marjolein Ebregt, Edward E. Carey

**Affiliations:** ^1^Department of Plant Science and Technology, University of Jos, Jos, Nigeria; ^2^Reputed Agriculture 4 Development Foundation, Kumasi, Ghana; ^3^International Potato Center, Kumasi, Ghana; ^4^CSIR-Savanna Agricultural Research Institute, Nyankpala, Ghana; ^5^International Potato Center, Nairobi, Kenya

**Keywords:** patrilineal, matrilineal, user orientation, awareness creation, value chains, training

## Abstract

Gender responsiveness in breeding programs to meet client and end user preferences for crops is essential. This case study analyzes the implementation experience of gender-responsive breeding and variety dissemination in Malawi and Ghana, focusing on good practices and challenges encountered. In Malawi, a training-of-trainers approach was employed to share knowledge among trained farmers. In Ghana, a research study was conducted to identify gender-based preferences for sweetpotato to define breeding objectives. The participation of social scientists, food scientists, and sweetpotato breeders in the GREAT (Gender Researchers Equipped for Agricultural Transformation) team provided a multidisciplinary perspective, addressing questions and responses in the field. Research efforts were strengthened by focusing on food quality through the establishment of an analytical laboratory for rapid evaluation of nutrition and food quality, including sugars. This helped develop sensory analytical capacity to better understand quality attributes and market segments, guiding breeding and improving market opportunities for women. Breeding outcomes resulting from gender inclusion led to the release of some sweetpotato varieties meeting end user and consumer preferences, as well as adoption of OFSP varieties by men and women. Other good practices for gender inclusion and responsiveness include providing funds for gender-based research and activities, engaging gender specialists and social scientists in trans-disciplinary teams, designing program activities with gender considerations, and incorporating traits in seed multiplication and dissemination decisions. Application of these gender inclusion practices resulted in adoption and development of acceptable sweetpotato varieties.

## 1 Introduction

### 1.1 Why sweetpotato breeding and dissemination efforts paid attention to gender in Ghana and Malawi

Sweetpotato has traditionally been considered as a women’s crop grown for food security in Sub-Saharan Africa (SSA). It has been increasing in importance in recent years, and now accounts for 33% of global production, up from 15% in 2009 (Food and Agriculture Organization of the United Nations, 2023). While this was partly due to declining production in China, which is still the largest global producer, both area and yield of sweetpotato have also been increasing across SSA during the same period. The importance of sweetpotato in food and farming systems varies across regions, ranking from among the most important crops in some countries such as Malawi, to having a relatively minor, though regionally important role in others such as Ghana. According to Food and Agriculture Organization of the United Nations (2023), in Malawi, sweetpotato ranks first in production and fourth in production area among crops. In Ghana it ranks twenty-first in production and nineteenth in area. *Per capita* annual production in Malawi is reported to be roughly 402 kg, while in Ghana it is 4.3 kg per person. As others have noted, FAOSTAT data for sweetpotato in SSA are quite unreliable, but they at least provide some point of comparison and may be improving as the crop becomes more mainstream (Andrade et al., 2009; Carey et al., 2021).

Traditionally, across SSA, sweetpotato storage roots have been eaten boiled, fried, or roasted. The predominant and preferred root quality types are staple types with white or yellow flesh, a relatively dry and mealy cooked texture and mild flavor. Sweetpotato tops have also been important as a leafy vegetable or animal feed, varying by region. Sweetpotato, along with other root and tuber crops, was for many years neglected by crop improvement programs since they were not of great commercial interest. However, sweetpotato research and development in SSA has picked up significantly in the last thirty years, driven by recognition of its high production potential, climate resilience, nutritional value, and potential for value addition (Woolfe, 1992; Grüneberg et al., 2015; Mwanga et al., 2017; Low and Thiele, 2020). The demonstration that orange-fleshed sweetpotato (OFSP) can help to combat the serious public health problem of vitamin A deficiency in children under five years old and pregnant and lactating women also contributed significantly to the efforts and have garnered much donor support (Low et al., 2017). These efforts have led to the strengthening of sweetpotato research and extension across SSA, including in Ghana and Malawi, the two countries reported on here.

Sweetpotato value chains have developed both through project interventions, using OFSP as an entry point, and informally, through natural strengthening of local and urban markets for staple-types for traditional uses. These markets provide demand for new varieties, seed delivery systems and services that research and extension programs and other service providers can respond to. Studies and reports by Sugri et al. (2017) and Peters (2015) described and made recommendations about value chains in Ghana. A similar study by van Vugt and Chibwana (2016) described and made recommendations for Malawi. Moyo et al. (2022) present a review of development and use of OFSP puree as an innovative ingredient in baked products, including in Malawi. As products and value chains diversify and strengthen, it is critical for service providers, such as breeding programs to be responsive to the requirements and preferences of the various actors, including women and children.

### 1.2 Gender relations in Sweetpotato in Ghana and Malawi

Sweetpotato was reported to be a women’s crop in the subsistence farming systems of Kenya and Uganda (Mutuura et al., 1992; Bashaasha et al., 1995). In contrast, sweetpotato, while mainly produced for home consumption, was produced by both men and women in Uganda (Abidin, 2004; Ebregt et al., 2004), Malawi (Sindi et al., 2013) and in Ghana (Amengor et al., 2015; Etwire et al., 2018). Amengor et al. (2015) reported that men primarily handle land preparation and planting, while women take charge of weed control, fertilizer application, harvesting, and marketing. Additionally, Adekambi et al. (2020a,b) reported that in northern Ghana, both men and women participate in planting sweetpotato vine cuttings, weeding, and hilling-up the fields. However, men usually sell the roots wholesale, as it is believed that they are better able to negotiate prices with traders and are strong enough to load their sacks of sweetpotatoes onto the trucks as requested by the traders. Women take charge of retail sales in local markets. Late in 2012, a study of the sweetpotato value chain in Nigeria, Ghana, and Burkina Faso noted that much of the sweetpotato in each country was being fried by women for local sale (Peters, 2015). In Ghana and Malawi, as elsewhere, women tend to do the household cooking, so they are highly focused on culinary quality.

In SSA, land tenure can be by matrilineal or patrilineal inheritance. In Malawi, matrilineal kinship is common in Phalombe and Dedza districts where the work described here was implemented. In Ghana, matrilineal kinship is found in the Ashanti Region where sweetpotato is not a major crop, however our project efforts reported here were mainly in patrilineal areas. In matrilineal communities, the land is owned by women or matrilineages and the husbands have a designated residence status. In Phalombe and Dedza in Malawi, a married man moves to his wife’s village. These married men are asked to work on the land if the household is engaged in agriculture to feed the family. Women help the men as already mentioned, with men doing some of the heavier tasks. Income is managed by both husband and wife for household needs, including school fees and maintaining the family’s house. About a third of the most fertile land is used to grow crops for household needs, whereas another third is used by the husband as he deems fit, and the last third is used by the wife (Sindi et al., 2013).

According to Quaye et al. (2016), in patrilineal communities in Ghana, men control over 80% of agricultural land, and women typically have poor access to land resources and money, and a minor voice in decision-making. The land is usually inherited by sons while daughters go to live with their husbands. The type of crops grown by women, the amount of land farmed, and its productivity are all impacted by women’s weak rights over agricultural lands. Additionally, poor markets hamper women’s engagement in farming alongside ineffective processing methods. In both patrilineal and matrilineal systems, family lands are kept in trust for posterity and are not transferred. Hence, deliberate efforts are needed to ensure that women as well as men benefit from breeding, variety dissemination and other efforts to improve the lives of all intended beneficiaries of efforts to improve livelihoods and strengthen value chains.

### 1.3 Context

The organizations and actors described in this paper were core actors in a number of projects in Ghana and Malawi under the Sweetpotato for Profit and Health Initiative (SPHI). The International Potato Center established its regional sweetpotato breeding platform for West Africa at the CSIR-Crops Research Institute in Ghana in 2010. Ghana’s national breeding program was chosen because of its strong institutional capacity, including strong support for root and tuber crop research and extension in Ghana at that time from both the World Bank, and the International Fund for Agricultural Development. The national sweetpotato breeding program in Malawi was backstopped from CIP’s regional breeding platform for southern Africa in Mozambique. OFSP was a primary, though not exclusive target of the efforts which had the overall aim of repositioning sweetpotato in the food economies of sub-Saharan Africa. Breeders were consistently engaged with these projects in each country and thus were positioned to understand and respond to feedback from all projects. This case study aims to document and analyze efforts to include gender in breeding, variety dissemination and other activities and projects carried out in Malawi and Ghana, drawing primarily on experiences of projects the authors were involved with under the SPHI.

## 2 Analysis

### 2.1 Research and other sources of information generated on gender

Prior to 2009, national variety release guidelines across much of Africa already had guidelines stipulating the need to demonstrate “value for cultivation and use (VCU)” (Setimela et al., 2009). This information typically involved the use-of farmer participatory on-farm trials and culinary evaluations, with women heavily engaged in the process of cooking and tasting. In both Ghana and Malawi, one of the earliest variety releases was of an early-maturing, widely adapted, yellow-fleshed farmers’ variety, originally from Tanzania, which had high yields and very good culinary quality (Otoo et al., 2000; Moyo et al., 2004). These user-oriented efforts effectively engaged both men and women in breeding assessments, though gender analysis was not an explicit element of the breeding and selection process.

Under the SPHI, starting in 2009, the International Potato Center (CIP) and partners focused on breeding new varieties and strengthening seed systems and value chains through various projects. CIP’s projects in sub-Saharan Africa (SSA), prompted by donor priorities, consistently targeted women as key beneficiaries (Abidin et al., 2024; Supplementary Table S1). National research programs, ministries of agriculture, and women’s development extension programs were actively engaged in these efforts. The SPHI emphasized the development, dissemination, and use of provitamin A-rich OFSP varieties to help combat micronutrient deficiency, which is a serious public health problem, especially for young children and pregnant and lactating women in much of SSA (Low et al., 2017; Low and Thiele, 2020). These efforts thus sought to combat micronutrient deficiency and improve livelihoods.

Under the SPHI, breeding was supported by the Sweetpotato Action for Security and Health in Africa (SASHA) project which established breeding support platforms in Uganda, Mozambique, and Ghana to backstop regional varietal development and dissemination efforts by national programs. The SASHA multi-disciplinary research team included breeders, seed specialists, postharvest experts, food scientists, and social scientists (economists and gender specialists) to ensure project success. These multi-disciplinary teams interacted regularly, bringing their perspectives and skills to specific project components and team meetings, contributing to the user orientation of breeding efforts and SPHI successes.

During stakeholder consultations and priority-setting exercises for the SASHA project, the critical importance of attention to gender was recognized since women were heavily engaged in smallholder production for household consumption and local sales, and pregnant and lactating women and their young children were the intended primary beneficiaries of efforts to develop and disseminate OFSP. At the breeding platform for West Africa in Ghana, the development of low-sweet, staple-type, varieties was also prioritized since sweetpotato, being sweet, was not considered a staple food, but mainly consumed as a snack. Developing non-sweet types would stimulate new demand for the crop, similar to yam, the regionally important staple (Andrade et al., 2009). Timely access to healthy planting material, postharvest perishability, virus, and weevils were also identified as key constraints along with poor market access. Thus, an integrated approach to breeding, variety dissemination, production and promotion was needed.

Aside from the low-sweet, staple types already mentioned, there was a recognition that consumer-accepted sensory quality attributes were essential, especially for OFSP varieties which were often moister, with lower dry matter content and stronger flavor than the traditional white and yellow-fleshed varieties. Newly introduced OFSP varieties thus ran the risk of consumer rejection. Selection of higher dry matter, drier textured OFSP thus became an important selection criterion. We also recognized that consumer demand can be modified through targeted messaging on health and market opportunities (including new forms of utilization). Planting material dissemination efforts also emphasized use of virus-free, or apparently healthy seed, since timely availability of clean seed is key for high yields (Clark et al., 2012).

The Rooting out Hunger in Malawi with Nutritious OFSP (ROH-OFSP) project took a holistic approach to improving the livelihoods of women while disseminating OFSP, and knowledge on its use and processing (CIP, 2013a,b,c; Abidin, 2014a,b). Strengthening the seed system was critical to ensure adoption. An OFSP variety, Zondeni, released in 2008 was promoted by the project (Chipungu et al., 2010). Links between breeding, seed system, and dissemination efforts in ROH-OFSP used a farmer-centered approach described by Abidin and Carey (2018). The seed system approach involved multiplying and having available true-to-type, healthy planting material at primary sites, and establishing decentralized multiplication sites managed by specialized vine producers (secondary multipliers) or root and vine producers (tertiary multipliers). The approach encouraged commercialization of planting material to generate income. The approach also involved strong partnerships, advocacy and awareness creation, combined with decentralized training on all aspects of production, nutrition, utilization, and strengthening market linkages. A similar approach was used by other projects including Jumpstarting OFSP in West Africa through Diversified Markets in Ghana, Burkina Faso, and Nigeria (Abidin and Carey, 2017), and two projects on development and dissemination of sand storage technology for preservation of sweetpotato roots for consumption and production of planting material in drought-prone areas.

During the ROH-OFSP project activities in Malawi, a gender specialist assisted to help understand why females were not being reached in the desired numbers during training activities. A study was conducted in 2013 in two districts, Phalombe, and in Chikwawa, with matrilineal and patrilineal communities, respectively. In Ghana, the Gender Responsive Researchers Equipped for Agricultural Transformation (GREAT) project provided an opportunity for a multidisciplinary team involved with the breeding program to be trained on methods to bring a gender lens to the program. The team included a breeder, an economist, and a food scientist. Male and female producers, traders and consumers were surveyed in two farming areas in northern Ghana where CIP projects had been operating. Roles and perceptions of male and female actors, attributes of leading varieties and attributes desired in new varieties were studied. Details of the gender studies in Ghana and Malawi are presented in Supplementary Table S2 and discussed below.

### 2.2 How attention to gender influenced breeding and seed dissemination efforts

CIP’s sweetpotato breeding platform for West Africa was established in 2010 in Kumasi at the CSIR-Crops Research Institute (CSIR-CRI). The CIP program worked with CSIR-CRI in the forest zone and CSIR-Savanna Agricultural Research Institute (CSIR-SARI) in the savannah zone of northern Ghana. Regrettably, CIP support for this platform ended at the end of 2020. Nevertheless, from 2010, breeding dossiers submitted to the National Variety Release Committee included information from multi-locational trials covering major agro-ecologies in Ghana and VCU information generated through farmer and consumer feedback on agronomic, postharvest, gender-differentiated culinary assessments (boiled, fried, pounded) and nutritional attributes. New varieties had superior attributes including improved planting material persistence, early yield, taste, and better shelf-life. Release documents demonstrated the user orientation, identifying user-preferred varieties for release and were also responsive to the potential for promotion of OFSP for health. Seventeen varieties were released between 2012 and 2020 (Ministry of Food and Agriculture, 2019; SARI variety release reports, 2019, 2020, unpublished).

In Ghana, the multi-disciplinary team under the GREAT project reported that male producers tended to focus on agronomic traits such as yield, early maturity (to sell fast), and resistance to drought, weevils, and diseases, while women mostly paid attention to culinary traits such as taste, short cooking time, dry matter content, vitamin A, and use of leaves as vegetables. Men’s and women’s preferences complemented each other and aligned with predominant roles of men and women in the production system. Men and women farmers wished for varieties which could store for a long time before deteriorating, had good yield, resistance to diseases, and were early maturing. Since the breeding efforts were developing high-yielding sweetpotato varieties with a range of quality attributes, there were no major failings in the breeding effort because of the failure of taking gender into consideration. The preferences of male and female consumers and traders were in general agreement (Bidzakin et al., 2019). However, the predominance in the Northern Region of Apomuden (an OFSP variety released in 2005), and in the Upper East Region of the white-fleshed farmer’s variety Obaari indicated the need for strengthening efforts to develop and disseminate new varieties. The GREAT study confirmed work of the Jumpstarting OFSP project from 2014–2017 which improved knowledge on utilization and processing, and sensitization on health benefits of OFSP. The survey results of the Jumpstarting project were published by (Adekambi et al., 2020a,b).

The ROH-OFSP project in Malawi attempted to target women in training on production, nutrition, processing and utilization, aimed at improving knowledge and skills at the farm household level so as to ensure sustained adoption. A training-of-trainers approach was taken with the expectation that trained farmers/agencies should share their knowledge with 5–10 other farmers. As already mentioned, women’s attendance was lower than expected. A gender survey in 2013 was carried out to understand why few females attended training events. Similar findings in Chikwawa (with patrilineal kinship) and Phalombe (with matrilineal kinship) suggested that women’s low participation was not related to gendered differences in land tenure. Rather, many women were shy to participate in training (CIP, 2013c, p. 14) because of limited formal education and relatively low self-confidence. They trusted their husbands to participate for them with the expectation that they could learn from them later. In worse cases, men forced women to stay at home so they could participate in project activities. It was also found that government extension agents, the project partners, mainly chose men to participate in trainings (Mudege et al., 2017, 2018). These insights helped improve the ROH-OFSP project’s efforts to reach women. Emphasis was given to increasing female participation in training and building the capacity to reach both husbands and wives by taking a household approach.

### 2.3 Methods and approaches: advantages and shortcomings

Partnerships and commitment by national agricultural research and extension services (NARES), and networking stakeholders are important to drive increased consumption, adoption, utilization, availability, and trade of sweetpotato among men and women. However, as illustrated by the trainings in Malawi, special efforts are needed to ensure that more women benefit than is often the case, particularly when commercial opportunities are involved. A recent example highlighting the need for more work to target women in Ghana was provided by a report on adoption and benefits of sweetpotato dissemination efforts by CSIR-CRI and the Ghana Ministry of Food and Agriculture under the West Africa Agricultural Productivity Programme (WAAPP). There was high adoption of improved sweetpotato varieties and production practices, but men benefitted much more than women (Acheampong et al., 2024).

**Figure 1:**
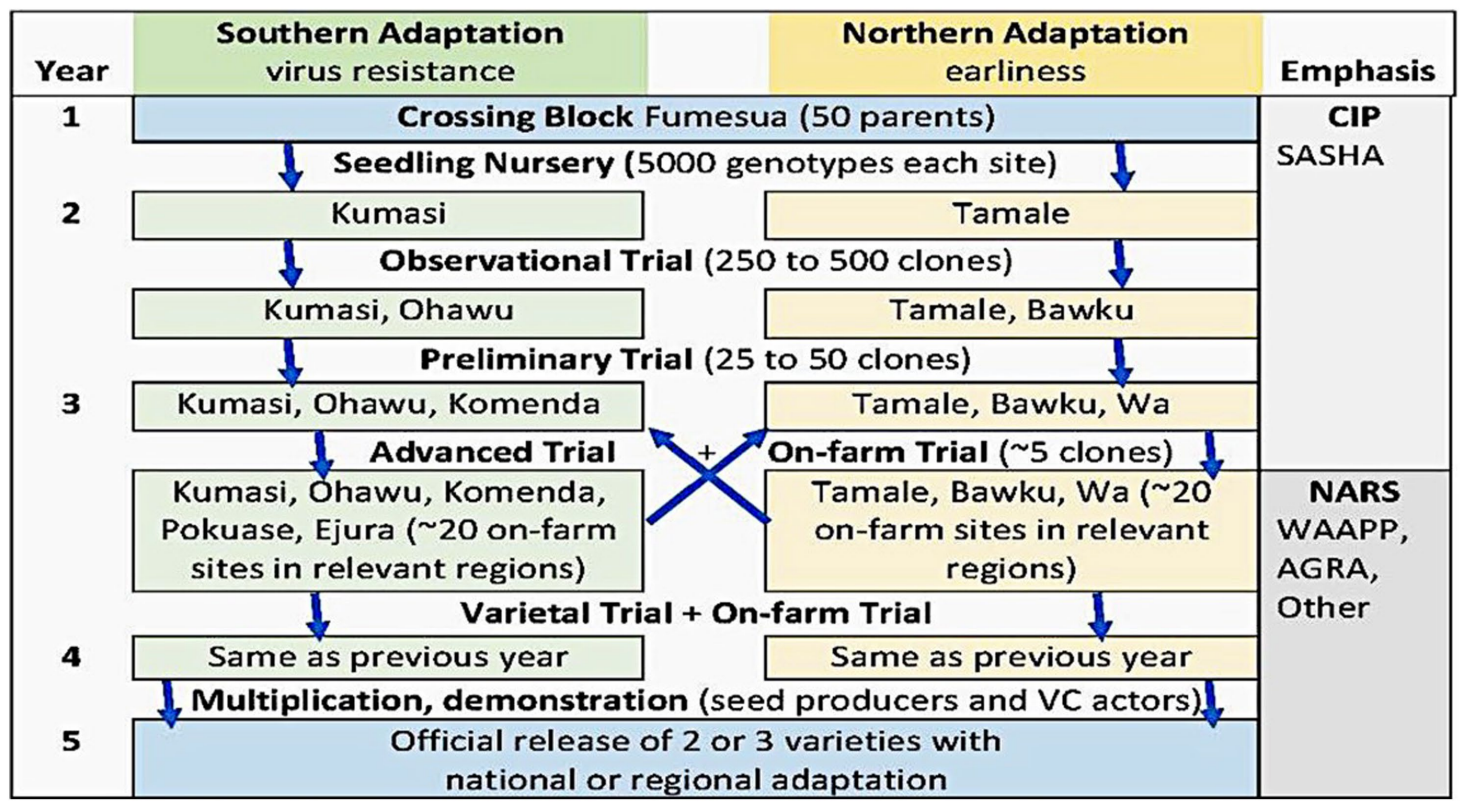
Accelerated breeding scheme in Ghana and West Africa with defined environments for population improvement in partnership with NARS.

The SASHA breeding platforms were equipped with near infrared reflectance spectrometers to assist with rapid screening of sugars, several minerals and total carotene in raw, freeze-dried sweetpotato roots. This assisted with quality screening of large numbers of early generation clones as part of an accelerated breeding scheme designed to lead to the release of new varieties in 4 or 5 years (Grüneberg et al., 2015). Figure 1 presents the scheme that was used in Ghana, evaluating selections at ecologically distinct CSIR-CRI and CSIR-SARI sites in the north and south of Ghana, and conducting on-farm trials in the third and fourth years. At the early stages of selection, traits such as pest and virus resistance, vine vigor, yield and root appearance were of necessity given priority over sensory attributes. Furthermore, sugars in raw sweetpotato roots are not a predictor of the sweetness of cooked roots since starch is hydrolyzed to maltose during cooking (Carey et al., 2019). So, while it was nice to have the ability to rapidly screen for traits such as sugar which is an aspect of culinary quality, it required additional efforts to enable us to predict quality attributes and consumer acceptance in cooked sweetpotato. Thus, the laboratory served as a nucleus for developing the sensory analytical capacity needed to better understand quality attributes and market segments and help guide breeding for different types of products (Dery, 2020; Dery et al., 2020; Ssali et al., 2020).

As already mentioned, on-farm trials were an essential element for farmer involvement in variety development and can provide input from consumers and other value chain actors in gender intentional ways. However, they often present challenges with respect to practicality and generating results that are easy to analyze and highly informative. The triadic comparison of technologies (tricot) is a citizen science method that provides a data-driven approach to on-farm and consumer sensory testing (de Sousa et al., 2024). The method uses an incomplete block design which allows individual farmers or consumers to compare varieties (or other technologies) in groups of three, by simply ranking them with regards to characteristics of importance. It is supported by a management and analytical platform that enables easy and highly informative analysis of results of these experiments and which can provide insights on gender, cultural and environmental effects, including generating recommendation domains within a country for the varieties being tested. With partners in Ghana, we used the tricot method to evaluate already released and pre-release selections from the breeding program by large-scale on farm and consumer sensory testing and showed gendered differences in taste preferences for boiled and fried sweetpotatoes (Moyo et al., 2021; Supplementary Poster 1). This new method will require efforts to encourage variety release committees, breeders, and extension partners to adopt this powerful new tool. Resources will be required for capacity development and its routine use, and to engage key actors in providing feedback to the breeding process.

### 2.4 What in the breeding process changed as a result of learning about gender

Gender sensitivity ensured the participation of relevant actors, including farmers, processors, and consumers, and the inclusion of men and women in evaluations and analysis of results. Selection for high dry matter was an obvious objective aimed at reducing oil absorption on fried OFSP and otherwise improving texture of boiled roots. This resulted in the breeding and release of new, higher dry matter OFSP varieties, such as SARI-Nan, SARI-Jan Low released by CSIR-SARI in Ghana in 2019 and 2020, and in the release of 8 new OFSP varieties in Malawi in 2011 and 2018 (Gatto et al., 2021).

Understanding the needs of and potential market segments for non-sweet and other types of sweetpotato was also very important, particularly since some were skeptical that development of this non-sweet sweetpotato would be useful. Development of sensory lexicons to describe the appearance, texture, flavor and basic taste attributes of fried and boiled sweetpotato roots was a first step. Sweetpotatoes characterized by trained sensory panels could then be evaluated in important sweetpotato production regions and markets by consumers providing their preferences for these varieties. These efforts revealed very different preference clusters differentiated by location, and age of participants, though not so much by gender. It was noteworthy that a preference for the non-sweet (staple-type) sweetpotato was identified in southern Ghana, while sweeter types were preferred in the north. It was also noteworthy that the higher dry matter, but sweet OFSP, SARI-Nan, was preferred by consumers in each location (Dery, 2020; Dery et al., 2020; Ssali et al., 2020). Such insights are essential for understanding and catering to the needs of these segments, while maintaining a consistent gender focus, to ensure equitable outcomes.

The development of a sand storage technique in Malawi and Ghana provided a tool for ensuring timely availability of planting material and enhancing the dissemination of sweetpotato varieties with a short shelf-life. Short shelf-life, and timely access to planting material are key constraints to increased adoption of sweetpotato (Andrade et al., 2009; Bidzakin et al., 2014). USAID-OFDA-supported projects that developed and disseminated a sand storage technique to extend the shelf-life of harvested sweetpotato roots, to help farm families overcome the constraint of poor seed availability, especially in dryer areas. While reducing postharvest perishability of sweetpotatoes is a breeding priority, this sand storage technique can extend shelf-life of varieties with a shelf life of one to 2 weeks, by several months. In Malawi, during the sand storage development, women farmers helped design a storage pit with steps that made storing and removing sweetpotatoes more convenient than other methods including sandboxes, grain silos, and pits without steps or other methods that had been tried. In both Ghana and Malawi, sand storage enabled women to use household level storage to feed their families or sell, and to sprout roots and quickly produce vines when they need to plant in response to the increasingly variable onset of rains due to climate change (Atuna et al., 2017; Abidin et al., 2018a,b, 2019).

### 2.5 Breeding and dissemination outcomes and impacts related to gender equity

Developing and promoting diverse varieties of both low and high dry matter sweetpotato, such as CRI-Apomuden, SARI-Nan, SARI-Jan Low, along with a purple-fleshed genotype rich in antioxidants, SARI-Diedi, effectively addressed the diverse requirements of various potential markets and user segments. Varieties with attributes such as high dry matter are often preferred by women sweetpotato fryers (Ssali et al., 2020) and nutrient rich varieties meet the nutritional preferences of both male and female consumers (Bidzakin et al., 2019). Newly released high-yielding yellow and white-fleshed staple varieties, favored by both consumers and processors (women fryers), have also made a significant impact. Additionally, officially released farmer varieties and the landrace, Obaare (CSIR-Nyumingre) have contributed to this dynamic landscape (Bidzakin et al., 2019; Acheampong et al., 2024).

Strengthening the market system through demand-led value chain intervention was the focus of a recent AGRA project in Ghana. This and other investments in Malawi and Ghana reflect the scaling of demand for OFSP emerging from previous efforts. These efforts also stimulated demand for gender-responsive sweetpotato breeding in both countries (Supplementary Table S1). It is essential, however, to continue to foster integrated efforts which intentionally target women beneficiaries through household-based approaches in order to improve both nutrition and incomes, and to encourage the development of feedback loops between breeding programs and the users they exist to serve.

### 2.6 Adoption and its impacts

The development of improved varieties, strengthened seed systems, promotional activities and value chain support accounted for increased adoption of sweetpotato varieties. The Jumpstarting-OFSP project (2014 to 2017) and the USAID-OFDA sand storage scaling project (2017 to 2018) familiarized women and men in project areas and beyond with the low dry matter OFSP variety Apomuden. The projects created awareness of OFSP utilization and where to buy cleaned planting material. By teaching consumers to fry and bake with sweetpotatoes in local markets, and in school feeding programs, Apomuden found a market niche; paving the way for newer OFSP varieties such as SARI-Jan Low, released by the breeding program. This effort with male and female participants contributed to food security, increased income, nutrition, healthy food systems, and food justice, as locally grown OFSP supplementing wheat-flour for baking has the potential to reduce reliance on imported wheat. This multi-year, multi-project effort created a demand for an OFSP that had previously been rejected because of non-preferred characteristics, opening the door for improved OFSP varieties bred to replace Apomuden (Adekambi et al., 2020a).

In Malawi, the ROH-OFSP project (2010 to 2014) focused on one OFSP variety, Zondeni in the beginning of the project intervention in 2010. It was a local variety, officially released by the National Research Program in 2008. The dry matter is relatively high with good taste, and easily disseminated because men, women, and children liked its taste. Awareness creation combined with information on nutritional value, and potential for generating income through food processing (Abidin, 2010, 2014a) encouraged the adoption of newer high-yielding OFSP varieties, disseminated using similar approaches to the ROH-OFSP project in subsequent projects (Gatto et al., 2023).

## 3 Discussion

### 3.1 Good practices

*Gender research budgets in projects.* Projects in Ghana and Malawi did not have specific operational budgets for gender research related to breeding, seed, or value chains, but were able to allocate these from overall program budgets, drawing on program staff and special programs when needed. For breeding programs, the key is to routinely engage gender specialists and social scientists, or to use appropriate tools, to ensure that input from relevant stakeholders contributes to decisions about product advancement and variety release and dissemination.

*Gender considerations in targeting market(s) or end-users*. This was driven by overall SPHI objectives of improving the livelihoods of households through improved health and incomes. Since women often prepare food at home, are mothers and primary caretakers of children, or work as food vendors their needs must be targeted. It is necessary, however, to target these users, who can be overlooked in farming systems where men dominate, as was the case for training in Malawi, and is even more the case in northern Ghana where conscious efforts must be made to include women.

Small-scale bakers and vendors of snacks such as *mandazi,* a fried dough in Malawi, and chunk fries in Ghana, are mostly women; they have benefitted extensively from project activities which generated new demand and markets for OFSP (CIP, 2013a). Training in Malawi and Ghana on techniques to reduce oil absorption during frying (e.g., parboiling, drying, or coating) also helped women tap into wider markets (Abidin et al., 2015).

*Gender and decisions about breeding objectives and the desired impact*. All project activities were designed with gender in mind, targeting women also as beneficiaries. Sweetpotatoes are a family crop, thus, men and women should benefit. Breeding and seed systems can be more responsive if they are demand-driven, and oriented toward specific user groups, with feedback loops between breeding programs and beneficiaries. Leaf quality was identified as important to women, but it did not become a specific breeding objective in Ghana or Malawi, because the leaves of most varieties are suitable for eating as a vegetable. A flyer for consumers on how to use sweetpotato leaves as a vegetable or in juice in Ghana and Malawi was published (Abidin, 2016).

*Gender and* var*iety design.* Desired traits for various uses are selected along with other characteristics required by farmers and other value chain actors. The breeding approach taken by SASHA in Ghana involved making crosses followed by evaluations and selections of clones for traits such as vine vigor, virus, and weevil resistance, root size, root shape, yield, shelf life, etc., in key environments in the North and South (Figure 1). While we could pay attention to quality attributes such as flesh color and dry matter content throughout the selection stages, it was not possible to engage in massive evaluations for processing or culinary attributes at the early selection stages. In Ghana we were interested in different quality types, including non-sweet, staple types, sweeter, high dry matter OFSP, and other flesh and skin colors. We refined these objectives during the time the platform was in operation, improving our understanding of potential market segments in the North and South of the country. As markets for processed products and export pick up, greater attention will be required for smooth, attractive root shapes that are easy to wash, and which are in line with consumer expectations in export markets. A large portion of sweetpotato in West Africa is consumed in the form of chunk fries, mostly processed, and sold by women as a “street food,” so breeding programs need to ensure suitability for this use if they are to provide varieties for this massive market (Carey et al., 2021).

Various varieties and advanced selections came through the breeding pipeline, providing high-yielding, culinarily diverse, and accepted genotypes. We used the tricot method for consumer evaluation of advanced selections by men and women of the boiled and fried sweetpotato. These quality assessments were conducted in a market setting and were complemented by on-farm trial evaluations with males and females, so final assessments of acceptability are based on both agronomic and culinary performance (Moyo et al., 2021; Supplementary Poster 1). There was gender differentiation in the ranking of both boiled and fried sweetpotato, with some of the advanced selections emerging as preferred by men and women. Some high dry matter, white- and yellow-fleshed selections were ranked highly by both men and women in boiled and fried forms. Men liked boiled OFSP CRI-Apomuden more than women did, perhaps because of their expectations of its health benefits.

*Gender and trait evaluation*. Product profiles guided the selection process. These are based on the needs of users, including women. Dry matter levels above 30% are preferred in all genotypes, though the low dry matter content OFSP CRI-Apomuden (under 24%) was successfully disseminated before superior new varieties were available. Dry matter content up to 35% and over is preferred for white- and yellow-fleshed genotypes, though there may be trade-offs if attributes such as appearance and yield are superior. Ultimately, the combination of attributes for growing, cooking, and eating sweetpotato must guide selection decisions, made by men and women end-users comparing experimental genotypes with currently important check varieties of each quality type.

*Gender and decisions about on-farm trials*. On-farm trials are an essential part of breeding evaluations but have been criticized for not producing easily analyzable data. The development of the tricot citizen science method overcomes this concern, providing informative, statistically-sound analyses based on gender-disaggregated preference ranking by a large number of farmers or consumers. Key factors that contribute to rank differences in preferences in trials are detected using the Plackett-Luce analysis of results (de Sousa et al., 2024). Tricot is a convenient, effective, citizen science method for engaging farmers, processors, and others in the evaluation of technical options, e.g., varieties. The tricot method is not yet routinely used in on-farm trials by the national breeding programs in Ghana and Malawi. However, efforts by CGIAR breeding programs to encourage its adoption are already gaining traction in some national programs. It is essential to ensure adequate budgets for this approach, which has been shown to be more cost effective than traditional approaches to on-farm testing (de Sousa et al., 2024).

*Gender and decisions about what types of farmers participate in evaluations*. On-farm evaluations typically involve men and women farmers, but any group of end-users can be engaged. Quality or processing assessments can also involve non-farmers. The tricot method allows analyzing environmental influences on crop performance as well as gender preferences.

*Gender and decisions about seed multiplication and dissemination*. This can be done appropriately, depending on the needs and opportunities, ensuring that required varieties are available for vine multipliers. These efforts should seek to strengthen business opportunities for women to help them in seed or root production and processing businesses, which would stimulate demand for planting material, as implemented successfully by our projects in Malawi and Ghana. There is a continuing need to strengthen the integration of breeding and seed system efforts.

### 3.2 Lessons

From 2010 through 2019, the SASHA breeding platform in Ghana worked with the national program and many other partners to develop and disseminate varieties. Together with other SPHI projects targeting users’ needs, these efforts contributed to improving the livelihoods of many households.

Efforts over the years contributed significantly to our understanding of and our ability to respond to needs in a gender-sensitive fashion. We refined our understanding of attributes required by value chain actors, from farm households to processors and consumers. Studies of consumer market segmentation enabled us to develop an understanding of potential markets for different quality types, including non-sweet, staple-type and sweeter types. The tricot method improved our capacity to understand and respond to users’ needs through on-farm trials and consumer taste tests. We hope the use of tricot will gain routine acceptance as a tool for breeding and seed programs. Capacity development and awareness creation efforts stimulated demand for sweetpotato (particularly OFSP) and encouraged processing, farming, and planting material businesses. In Ghana, the breeding program developed higher-yielding varieties with a range of quality types adapted to the dryer northern environments, and the more humid southern regions of the country. A sustainable program for producing and selling healthy planting material was established at the CSIR-CRI to supply foundation seed to producers, NGO projects, and seed multipliers. Dedication and resources will be needed to continue and to strengthen these efforts.

A new project to empower women in poor areas of northern Ghana is working with CARE-Ghana, and the Ministry of Food and agriculture to use various technologies, including high-yielding new varieties like SARI-Jan Low for vine multiplication, production, and processing (Temesgen and Gyan-Bassaw, 2024). Integrated grassroots projects will allow women to become more active users of the improved varieties, providing feedback to breeders on the need for further improvement. Selection of smooth and regularly shaped roots should be included to increase demand by markets. The unevenly shaped varieties previously released are not attractive nor easy to be cleaned for processing.

In Malawi, the ROH-OFSP project in partnership with the national breeding program and various government and international organizations like the United Nations Food and Agriculture Organization (FAO), non-profit and private-sector actors, laid a strong foundation for continuing dissemination and adoption of sweetpotato, including OFSP. This effort has continued with persistent national, international, and private-sector initiatives, enabling the release and dissemination of several OFSP varieties by the national program. Malawi is a leading producer of sweetpotato in SSA, as a result of the foundational efforts of the ROH-OFSP project and its integrated approach to strengthening seed systems and nutrition education.

The GREAT project, coming towards the end of the breeding and seed dissemination efforts in Ghana, helped confirm traits and varietal preferences of men and women value chain actors in northern Ghana. Women’s preferences were largely related to nutritional benefits and culinary use of sweetpotato while men’s preferences were predominantly in line with yield and marketability. The interests of men and women were mostly complementary, indicating the importance of ensuring the contributions of both sets of stakeholders during variety evaluations and surveys. The work was conducted in two different regions of northern Ghana with quite different market opportunities, which had a bearing on the varieties being grown, and the responses of men and women in these survey areas.

The participation of a social scientist, a food scientist, and a sweetpotato breeder in the GREAT team provided a multidisciplinary perspective, ensuring that their questions were addressed in the field. All team members participated in the GREAT training, ensuring that the research was gender-responsive. GREAT attached a mentor to each group, to guide the team members through the research. Mentors provided literature, and online meetings, and reviewed research documents.

Our recommendation to breeders interested in impact-oriented, gender-sensitive variety development is to embrace the trans-disciplinary nature of demand-led plant breeding and to use the increasing array of available tools such as defined gender responsive product profiles and tricot. Extension agents and project managers also play a critical role in providing feedback to breeding programs and they should also be brought onto the breeding and dissemination teams to ensure impact. Good communication and cooperation among disciplines and team members is essential. Methods for promoting new products, such as OFSP varieties, must pay attention to diversified market opportunities. Engaging with users will ensure that varieties are developed that can be integrated into equitable and sustainable food economies. Fragmented and sporadic project funding and changing institutional priorities may always present challenges to breeders. However, breeding and seed dissemination programs that serve a large population or important markets will be easier to justify economically in the long run.

## Data availability statement

The original contributions presented in the study are included in the article/Supplementary material, further inquiries can be directed to the corresponding author.

## Ethics statement

Ethical approval was not required for the study involving humans in accordance with the local legislation and institutional requirements. Written informed consent to participate in this study was not required from the participants or the participants’ legal guardians/next of kin in accordance with the national legislation and the institutional requirements. However, written informed consent was routinely obtained prior to initiation of all surveys reported here.

## Author contributions

OU: Writing – original draft. Writing – review & editing. PA: Writing – original draft, Writing – review & editing. ED: Writing – review & editing. JB: Writing – review & editing. NM: Writing – review & editing. ID: Writing – review & editing. ME: Writing – review & editing. EC: Writing – original draft, Writing – review & editing.
