# Supplementary material for: Gender mainstreaming in sweetpotato breeding and dissemination in Ghana and Malawi
Source: Front Sociol. 2024 Apr 30;9:1263438. doi: 10.3389/fsoc.2024.1263438 (PMC11092908; doi:10.3389/fsoc.2024.1263438)
Supplement: Supplementary file 1 [file Table_1.docx]

**Supplementary Table S1.** Sweetpotato for Profit and Health Initiative and scaling projects in Malawi and Ghana

| **Projects, PI, PMs, Donors^[[1]](#endnote-1)^** | **Organizations and projects** | **Countries, Regions** | **Actors involved including Breeders** | **Target beneficiaries** | **Gender-relevant reports and documents** |
| --- | --- | --- | --- | --- | --- |
| **A. Sweetpotato for Profit and Health Initiative Projects** | | | | | |
| **Project name:** Sweetpotato Action for Security and Health in Africa (SASHA) phase 1 and 2 (2009 – 2019). | | | | | |
| **PI:** Dr. J. Low, economist  **PM** for West Africa: Dr. E.E. Carey, plant breeder  **Funded by:** BMGF | **Leader:** International Potato Center (CIP)  **Direct partners:** CSIR-Crops Research Institute (CRI) and -Savanna Agricultural Research Institute (SARI)  **Indirect partners:** Farm Radio International, Ghana Ministry of Food and Agriculture//Women in Agricultural Development | **Ghana** Burkina Faso, Nigeria  **Regions in Ghana:** Northern, Upper East, Upper West, Central, Ashanti, Volta, Greater Accra | **Project Activities:** Breeding and seed system linkages  **#breeders:** CIP, CRI and SARI breeders (3)  **#social scientists and economists:**  CIP global: 5  NARS: 1  **#gender research:**  CIP global: 3 | The SASHA project sought to develop the capacities, products, and methods to reposition sweetpotato in food economies of sub-Saharan African countries to alleviate poverty and undernutrition, particularly among poor women and children. Key features: Targeting women from a nutrition perspective was expanded with a full analysis of the gender power relations that underpin use of the crop.  Projects with development orientation, downstream impact required on incomes and welfare of women as well as men, emphasis on making sure there were functional, gender-responsive seed systems | [CIP (2020](https://mel.cgiar.org/reporting/downloadmelspace/hash/1761d29c2bb40d61afaad18c32a17988/v/4e6bf7660ea9d12ffba38af8ff8fd379)) |
| **Project name:** Rooting Out Hunger in Malawi (Oct 2009 – June 2014) | | | | | |
| **PI/PM:** Dr. P.E. Abidin sweetpotato seed system scientist; PhD sweetpotato breeder  **Funded by:** Irish Aid Project in Malawi | **Leader:** CIP  **Direct Partners:** Department of Agricultural Research Services (DARS), Millenium Villages Project (MVP), Concern Universal (CU), Catholic Development Commission in Malawi (CADECOM)-Chikwawa, Ministry of Agriculture Department of Extension Services  **Indirect partner projects:** USAID-CU, MoA, Min of Gender and Nutrition/SUN 1000 Days Movement, WALA-CRS, USAID-OFDA Sand Storage project, FAO, Save the Children in Africa, Concern Worldwide, Universal Industries, Farmers Union of Malawi, Local company-Kasungu, Kachele Women Club & Irish-Malawian NGO in Salima | **Malawi**  **Regions: Southern** (Zomba, Chiradzulu, Phalombe, Mulanje, Ntyolo, Chikwawa, and Nsanje), **Central** (Balaka, Dedza and Salima), and **Northern** (Dowa, Kasungu, Mzimba, and Karonga) | **Project Activities:** Seed systems, dissemination strategies through vouchers and commercialization, linkage with CIP breeding, ToT OFSP processed products: local recipes, bread, juices from leaves and roots to generate incomes at household targeting women.  **#breeders:** 1 CIP-PI & 1 DARS  **#gender research:**  CIP global: 1  **#social scientist:**  CIP global: 2  CIP local staff: 2 | (1) Seed producers/multipliers (DVMs) based on gender lens; (2) Vulnerable beneficiaries: children below 5, women, and people living with HIV and cancer (total: 190,000 households grew OFSP at project end; (3) NGOs, FAO, entrepreneurships on vines and roots. (4) Small-scale entrepreneurs for processed products; (5) Government of Malawi for SDGs | Abidin ([2010](https://hdl.handle.net/10568/140491); [2014a](https://hdl.handle.net/10568/140490); [2014b](https://www.sweetpotatoknowledge.org/wp-content/uploads/2016/04/Malawi-Rooting-Out-Hunger-4pages-Flyers-AUG-2014.pdf))  [CIP (2013c)](https://cipotato.org/wp-content/uploads/2014/06/Annual-Report-2013-1.pdf)  Baseline survey ([Sindi et al., 2013](https://cgspace.cgiar.org/items/06c811df-44ce-4c86-8fa0-48b7e9830b49))  Mudege et al. [(2017](https://www.tandfonline.com/doi/full/10.1080/0966369X.2017.1383363)) Mudege et al. [(2018)](https://www.tandfonline.com/doi/full/10.1016/j.njas.2018.05.003) |
| **Project name: Jumpstarting orange fleshed sweetpotato in West Africa through Diversified Markets (April 2014 – May 2017)** | | | | | |
| **PI:** Dr. E. E. Carey  **PM:** Dr. P. E. Abidin  **Funded by:** BMGF | **R4D Initiatives leader:** CIP  **Direct Partners:** CSIR-SARI & CRI, KNUST, UDS, ACDEP, MoFA & Women in Agriculture Development (WIAD), Ghana Health Service (GHS), iDE Ghana, Ghana School Feeding Program, Local Governments, Partnership for Child Development (PCD)  **Innovation Network at Scale:** MEDA, CSIR-FRI, Farm Radio International, USAID-RING & SPRING, WFP, District Assembly, GASIP, Casa de Ropa, Kofi and Nane Annan | **Ghana,** Burkina Faso and Nigeria  **Regions:** Northern, Upper East, Upper West, Volta, Central, Eastern, and Ashanti | **Project Activities:** Seed systems and dissemination Strategies for commercialization through various markets, promotion of new products from low DM OFSP include OFSP purée, local food & bakeries (mostly women), caterers (women) for school feeding programs, created market systems and OFSP value chains. **#breeders:** CIP 2, SARI 1 & CRI 1, total of 4  **#social scientist:** 1  **#food scientists and nutritionists:** KNUST 1, GHS 1, UDS 1 | (1) Commercial Seed producers (DVMs); (2) Commercial root producers, (3) Children below five of age, pregnant women, lactating mothers, and household with children below five, (4) Marketers/Aggregators, (5) Food vendors, (6) Caterers for GSFP, (7) local markets (non-structured markets) and structured markets (through the government development program in the countries, (8) Bakeries, and (9) I-NGOs, such as Canadian-MEDA, USAID-RING-SPRING. Total: 117,231 HHs benefitted from OFSP from three countries, but Ghana >50% out above figure. | [Abidin and Carey (2017)](https://hdl.handle.net/10568/139959)  Adekambi et al. [(2020a)](https://journals.sagepub.com/doi/10.1177/0030727020950324)  [Adekambi et al. (2023)](https://www.tandfonline.com/doi/full/10.1080/20421338.2021.2015172) |
| **Project name:** Breaking postharvest-bottles: long-term sweetpotato storage in adverse climates (2013 - 2015) | | | | | |
| **PI:** Dr. Carey (**Ghana**) & Dr. Abidin (**Malawi**)  **Funded by** USAID-OFDA (**Ghana & Malawi**) and Irish Aid (**Malawi**) | **R4D Initiative/Lead:** CIP  **R4D Partners: In Ghana:** CSIR-SARI, MoFA, Catholic Relief Services (CRS), Famers' Community base and UDS-Nyankpala. **In Malawi:** CRS, Cadecom-Mzimba, MoA, Farmers' Community-base and DARS of Northern Region, Extension Depart.  **Networking Umbrella:** SPHI  **Innovation Network at Scale: In Ghana:** UDS-Nyankpala and USAID-RING. **In Malawi:** FAO-Kasungu, Farmers Union of Malawi in Dowa, DARS at Bvumbwe, Southern Region. | **Ghana & Malawi**  **In Ghana:** Bawku Municipal and Navrongo District (UER), and Nyankpala-Tolon district (NR). **In Malawi:** Mzimba and Kasungu districts (NR) and Bvumbwe Research (SR). | **-Project Activities:** Seed systems, breeding linkage, dissemination, Postharvest handling through sand storage technique, OFSP added values, market opportunities, and sweetpotato value chains.  **#breeders:** 2  **#social science and marketing specialists:**  CIP local staff: 2 | (1) Relevant government agencies, (2) root producers, (3) food vendors, (4) small-medium scale entrepreneurs, (5) farmers in drought-prone areas for food and nutrition security, (6) NGOs & organization (local and international). | [Abidin (2017](http://www.sweetpotatoknowledge.org/wp-content/uploads/2017/10/PRES08-ABIDIN-EXTENDING-ACCESS-TO-SP-ROOTS-USING-STEPPED-PIT-STORES-AND-SAND-BOXES.pdf))  [Abidin et al. (2016)](https://www.davidpublisher.com/Public/uploads/Contribute/57b56509a9997.pdf) |
| **Project name:** Extending OFSP availability for vulnerable households through good agricultural practices and postharvest storage (2017 - 2018) | | | | | |
| **PI:** Dr. P.E. Abidin  **Funded by** USAID-OFDA | **R4D Initiative/Lead:** CIP  **R4D Partners:** CSIR-SARI, MoFA, Famers' Community base, Local Government, MoFA.  **Innovation Network at Scale: In Ghana:** RTB-Scaling Project - TRIPLE-S PLUS, MEDA, ENVAC-WFP-MoFA, Farmers' Community base in Navrongo and Bawku. **In Burkina Faso:** Hellen Keller International (HKI) in Ougadougou | **Ghana** & Burkina Faso  **In Ghana:** Bawku Municipal and Navrongo District (UER), and Nyankpala-Tolon district (NR). **In Burkina Faso:** border communities with Ghana, in Navrongo and Bawku. **At Scale**: in Burkina Faso under HKI | **Project Activities:** Seed systems, breeding linkage, dissemination, Postharvest handling technology, OFSP added values, market opportunities, value chains and combating climate change on its uncertainty.  **#breeders:** 2  **#IT & M&E Specialist:**  CIP local staff: 1 | (1) Relevant government agencies, (2) root producers, (3) food vendors, (4) small-medium scale entrepreneurs, (5) farmers in drought-prone areas for food and nutrition security, and combating climate uncertainty, (6) NGOs & organization (local and international), (7) broad opportunity at scale on the UN-Climate Change and Conversion funding project. | Abidin et al. [(2018a](https://www.researchgate.net/publication/327968290_A_guide_to_storage_of_fresh_sweetpotato_in_sand_pits_or_boxes_Extending_fresh_sweetpotato_root_availability_in_drought-prone_areas_after_harvest); [2018b](https://www.researchgate.net/publication/341742275_P13_Putri_Ernawati_Abidin_Poster_Simple_but_Effective_Sand_Storage_Technology_NXPowerLite_Copy))  Abidin et al. ([2019](https://www.actahort.org/books/1251/1251_30.htm)) |
| **Project name:** 'Citizen Science, Triadic Comparison of Technologies (TRICOT) for Scaling (2020 – 2021). | | | | | |
| **PI:** Dr. Jacob van Eten (CGIAR-Bio-versity International)  **PM R4D Partner in Ghana:** RA4DF: Dr. Carey and Dr. Abidin  **Funded by** RTB Scaling project | **R4D Initiative/Lead:** CGIAR-CIP & International Bioversity  **R4D CIP-Partner to lead in Ghana (Oct 2020 to Dec 2021):** Reputed Agric 4 Development Foundation (RA4D)  **R4D Partners in Ghana:** CSIR-SARI & CRI, MoFA/WIAD, Regional MoFA & Extension  **Innovation Network at Scale:** Extension Agents, Farmers, NGOs and relevant government agencies with respect to Variety Released Committee. | **Ghana &** Rwanda  **8 Regions in** Northern and Southern Ghana. | **Project Activities:** Breeding and seed System linkage, market opportunities, climate change and uncertainty.  **#breeders:** CIP 2, & NARs 2, total of 4  **#IT, M&E, social scientists:**  **CIP global:** 3  **CIP local staff:** 1 | (1) NARs, Target farmer beneficiaries, (2) NGOs, (3) larger-scale private sector farmers, (4) Independent farmers contacted in markets, (5) schools, and (6) Department of Agriculture extension workers. | de Sousa et al., [(2024)](https://doi.org/10.1007/s13593-023-00937-1) |
| **B. Project at Scale under Scaling Partners** | | | | | |
| **Project name:** Demand Creation and Impact Scaling (DCIS) for OFSP in Ghana (2021 - 2022) | | | | | |
| **PM Scaling Partner:** Reputed Agric4Dev Foundation management team, i.e. Carey and Abidin  **Funded by** AGRA-OFSP for Scaling in Ghana | **Scaling project partner/Lead**: Kofi Annan Foundation (KAF) and Reputed Agric4Dev Foundation (RA4DF)  **Other Scaling Project Partners:** CSIR-CRI, Fresh Logistics, E-Darkey Ltd, Ohumpong Ltd, DKCL, Veronica Ltd  **Innovation Network at Scale:** MoFA/WIAD, MESTI Maphlix Trust Ghana Ltd, SAYEX & KAZ Ltd, Casa de Ropa, GHS, GSFP, Min of Technology and Investment (MoTI). | **Ghana**  **Regions:** Volta, Bono East, Eastern, Ashanti, Central Regions and Greater Accra | **Project Activities:** Breeding and Seed System Linkages, business model through creating market ecosystems through strong partnerships.  **# breeders:** Reputed A4D 2, CRI 1, total of 3  **#IT and M&E**: 1 (Reputed A4D)  **#social scientists:** 2 (KAF)  **#gender rights lawyer:** 1 (Mrs. Nane Annan - KAF) | (1) Global organizations and donor communities (by Mrs. Nane Annan of KAF), (2) OFSP value chain actors in all districts including the relevant government agencies and Local private companies (by RA4DF), (3) vine multipliers and root producers in value chains of Ashanti, Eastern, Central and Great Accra (by CSIR-CRI); (4) Local private companies, (5) Government agencies on SDGs | [RA4D (2022)](https://www.youtube.com/watch?v=-EPi8aM-ISU) |

1. PI – principal investigator, PM – project manager [↑](#endnote-ref-1)
