# Supplementary material for: Gender mainstreaming in sweetpotato breeding and dissemination in Ghana and Malawi
Source: Front Sociol. 2024 Apr 30;9:1263438. doi: 10.3389/fsoc.2024.1263438 (PMC11092908; doi:10.3389/fsoc.2024.1263438)
Supplement: Supplementary file 2 [file Table_2.docx]

**Supplementary Table 2.** Gender research activities, timeline, and how the data were used

| **Country** | **Role in Gender Research** | **Data collected** | **When?** | **Why?** | **How the data were used** |
| --- | --- | --- | --- | --- | --- |
| **Ghana** | Sweetpotato value and demand study for Ghana, Nigeria and Burkina Faso | Rapid rural appraisal methodology | Late 2012 to early 2013 | To clarify needs and opportunities for sweetpotato breeding and variety dissemination efforts in the countries targeted | Typologies of farmers based on farm sizes and market linkages were developed in each country. Gender was not explicit, but recommendations were based on recognition of actors’ capacities and roles. This was one of the first studies to recognize the importance of chunk fries as a major form of use.  [Peters (2013)](https://www.fao.org/sustainable-food-value-chains/library/details/en/c/383008/) |
|  | GREAT program. Design of gender research tools, data collection, analysis Project conceptualization and monitoring | Qualitative data using Focus Group Discussions, Quantitative data using individual surveys  Data analysis | April 2017 | To understand sweetpotato trait and varietal preferences by men and women Value chain (VC) actors which drive and shape varietal adoption  To help re-define breeding objectives for gender responsiveness and inclusion in varietal development | Partially reported in [Bidzakin et al. (2019).](https://hdl.handle.net/10568/140656) |
| **Malawi** | Project conceptualization and monitoring,  Design of gender research tools, data collection, analysis | Qualitative data using Focus Group Discussions, Quantitative data using individual surveys | 2013 | To understand sweetpotato trait and varietal preferences by men and women value chain (VC) actors which drive and shape varietal adoption through seed systems and dissemination programs  To understand the bottleneck on the gender equity within the ROH OFSP project areas in Malawi with the differences of matrilineal inheritance in Phalombe and patrilineal inheritance in Chikwawa | As part of Project Report to Irish Aid in Malawi  Publications:  [Mudege, et al. (2017)](https://www.tandfonline.com/doi/full/10.1080/0966369X.2017.1383363)  [Mudege et al. (2018)](https://www.tandfonline.com/doi/full/10.1016/j.njas.2018.05.003) |
