# Supplementary material for: Gender mainstreaming in sweetpotato breeding and dissemination in Ghana and Malawi
Source: Front Sociol. 2024 Apr 30;9:1263438. doi: 10.3389/fsoc.2024.1263438 (PMC11092908; doi:10.3389/fsoc.2024.1263438)
Supplement: Supplementary file 3 [file Presentation_1.pdf]

CITIZEN SCIENCE (TRICOT) EVALUATION OF SWEETPOTATO VARIETIES IN GHANA

Edward Carey[1], Kwabena Acheremu[2], Erna Abidin[1], Kwadwo Adofo[3], Moses Hadjor[2], Daniel Akansake[1], Joseph Adjebeng-Danquah[2], Joseph Awoodzie[3], Asimah Razak[1], Simon Imoro[1], Jacob Ulzen[4], Kauê de Sousa[4], Brandon Madriz[4], Reuben Tendo Ssali[5], Thiago Mendes[5], Jacob van Etten[4]

[1]Reputed Agricultural 4 Development Foundation
[2]CSIR-Savanna Agricultural Research Institute
[3]CSIR-Crops Research Institute
[4]The Alliance of Bioversity International and the International Center for Tropical Agriculture (CIAT)/One CGIAR
[5]The International Potato Center (CIP)

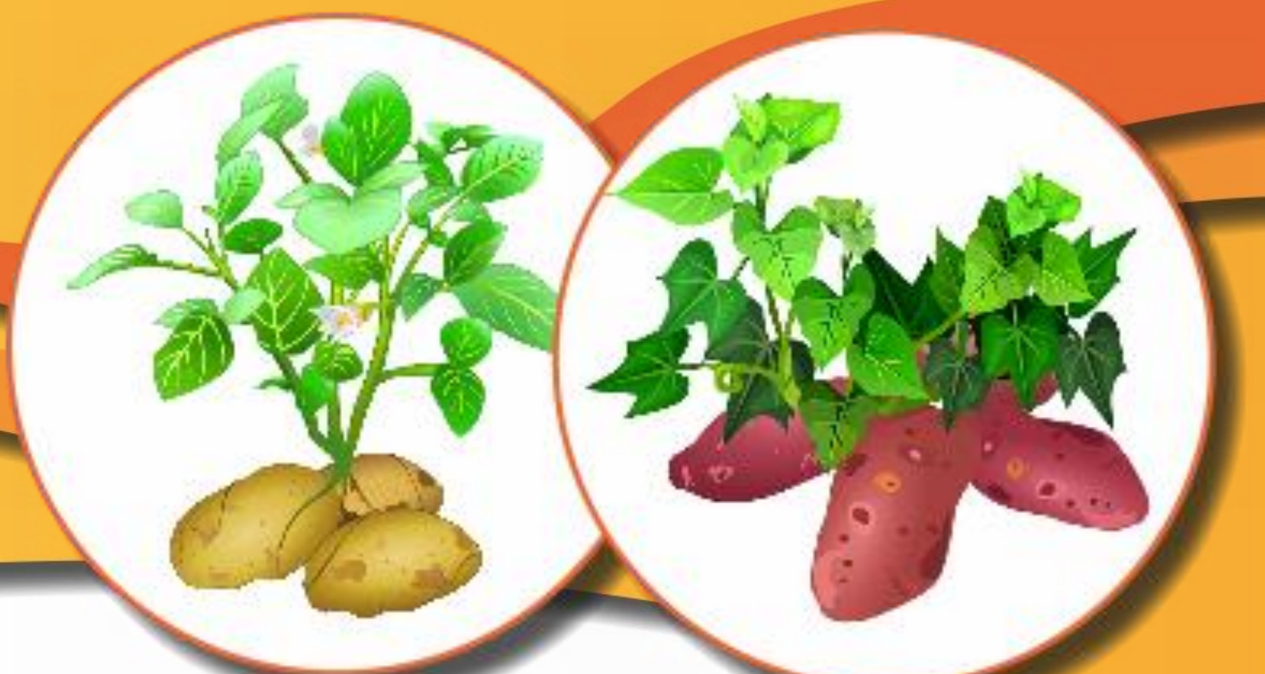

Abstract

The triadic comparison of technologies (tricot) method is a participatory, citizen science method for evaluating a number of technologies (e.g. varieties) in groups of three, with analysis based on ranking. The tricot method is supported by the ClimMob platform (ClimMob.net) which enables the design of on-farm experiments, collection of data (using ODK Collect), and analysis of results. The same approach can be used for consumer sensory analysis to complement the results of on-farm evaluations. During 2020 and 2021, the tricot method was evaluated in Ghana using recently released sweetpotato varieties and advanced selections from breeding programs at CSIR-CRI and CSIR-SARI. In 2020, sweetpotato evaluations were conducted with extension partners on 1549 farms across 7 regions in the north and south of Ghana, with final data collected from 915 farms. Results were analyzed and reported back to extension partners. In 2021, 1832 evaluations were conducted with 26 partners across 8 regions in northern and southern Ghana. In 2021, types of partners engaged in tricot studies were expanded to include NGOs, larger-scale private sector farmers, independent farmers contacted in markets, and schools, in addition to Department of Agriculture extension workers. Results from 2020 and 2021 trials, generated using the ClimMob platform, and results from combined analysis over trials using R to evaluate and predict the performance of genotypes across agro-ecological zones will be presented. We will also present results from consumer sensory assessments performed in market settings. The tricot approach was relatively easy to implement, allowing implementing partners, farmers and consumers to participate in the generation of robust results which can be used to refine recommendation domains, and justify the release of new varieties. With a body of research and extension partners familiar with the approach, we expect that tricot will be used increasingly in the future.

Introduction + Study objectives

- Participatory, on-farm research is critical to the development and dissemination of new agricultural technologies, including varieties, agronomic practices, etc.
- Typically, on-farm trials are cumbersome and do not result in highly informative results (Misiko, 2013).
- The tricot method uses a citizen science approach to involve many farmers or other end-users to rank technologies in presented in groups of 3 (using an incomplete block approach), producing highly informative results.
- We evaluated 17 recently released and advanced varieties in on-farm and consumer sensory trials with various types of partners in Ghana over a two year period with the objectives of a) refining recommendation domains for released varieties and gaining user feedback on pre-release varieties and b) gaining insights on which partners were best to work with for reaching many farmers in the most cost effective fashion.

Material and Methods

- The ClimMob platform (ClimMob.net) was used to randomize entries in incomplete block trials, assist with data collection (using ODK Collect App), analysis of data and reporting of trial results (van Etten *et al.,* 2020; de Sousa *et al.,* 2022)
- Standard questions to farmers varied somewhat from year 1 to 2 as we refined our approach.
- Research was implemented in collaboration with CSIR sweetpotato sweetpotato breeders and trials implemented with Regional Department of Agriculture (both years). In 2021, schools, large farmers using outgrowers, local NGOs, market sales and Department of Agriculture were used to reach farmers for trialing.
- Results were collected from farmers by direct or telephone interviews.
- Consumer sensory evaluation of boiled and fried sweetpotato was conducted in market settings in the North and South in 2021. Additional exotic genotypes were included in the evaluations.
- Feedback reports were generated for farmers to inform them of their individual results and overall trial results

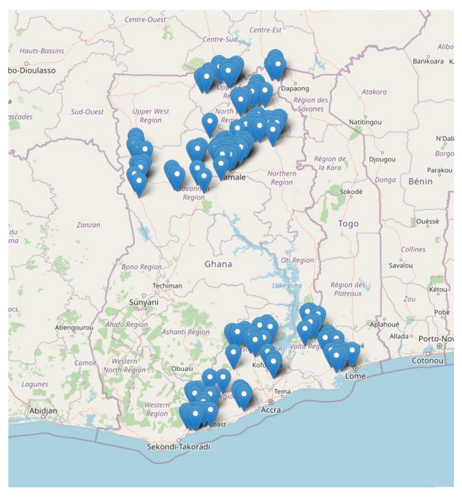

Trial locations in Ghana

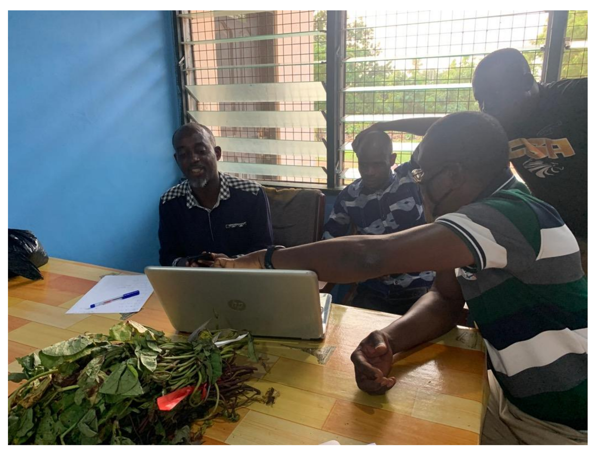

Orientation of Dept. of Agriculture

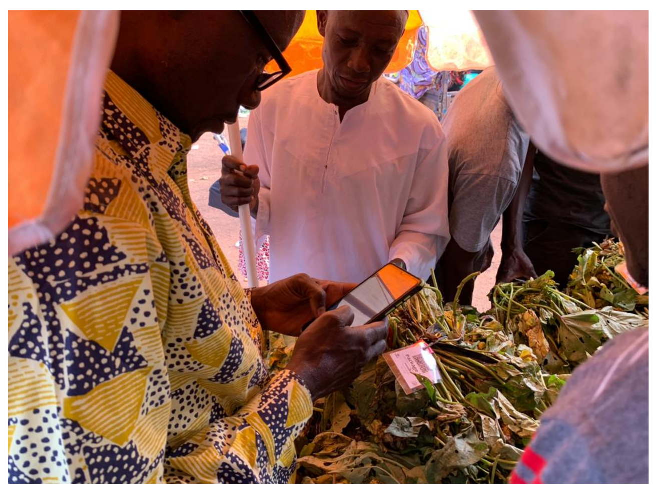

Sale of tricot packets at market

Table. Genotypes evaluated in trials in Ghana over 2 years

Genotype name	Variety status	Key attributes
CRI-Apomuden	Released	Orange flesh, low dry matter, high yield, widely adapted
CRI-Dadanyuie	Released	White flesh, high dry matter, high yield
CRI-Ligri	Released	White flesh, good storage, widely adapted across Ghana
Okumkum	Released	White flesh, high dry matter, high yield, excellent taste
PG17265-N1	Pre-release	Cream flesh, high dry matter, weevil resistant
PG17305-N1	Pre-release	White flesh, high dry matter, high yield.
PG17362-N1	Pre-release	Orange flesh, moderate dry matter, high yield.
PGA14008-15	Pre-release	Cream flesh, drought tolerant, virus resistant, southern-adapted (northern adaptation not known).
PGA14011-13	Pre-release	Cream flesh, drought tolerant, virus resistant, southern-adapted (northern adaptation not known).
PGN16021-39	Pre-release	Orange flesh, vigorous vines (dual purpose), high yield
SARI-Diedi	Released	Purple flesh, high dry matter, weevil resistant, moderate yield
SARI-Janlow	Released	Orange flesh, early maturing, dual-purpose, high yield
SARI-Nan	Released	Orange flesh, attractive root shape
SARI-Nyoriberi-gu	Released	Light orange flesh, moderate dry matter, high yield, widely adapted across Ghana
SARI-Nyumingre	Released	White flesh, high dry matter, high yield, commercial variety in Upper East
SARI-Suyolo	Released	White flesh, good storage
SARI-Tiemeh	Released	White flesh, high dry matter, high yield, good taste

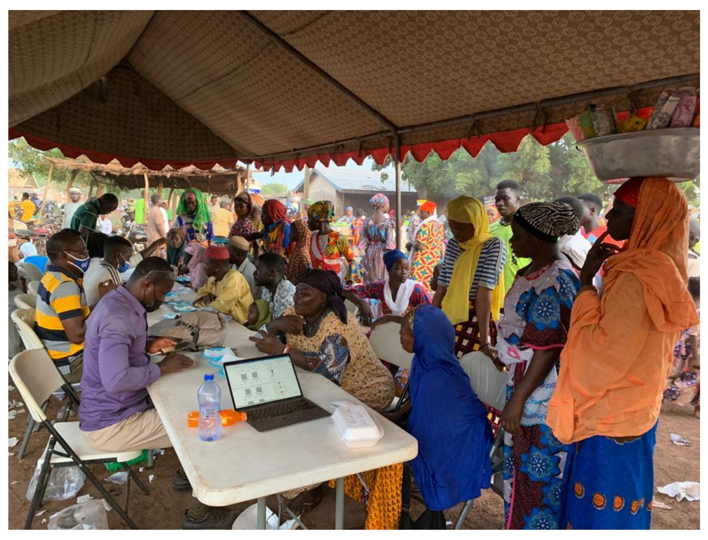

Sensory assessment of sweetpotato at market

Results and Discussion

Combined results from 2020 and 2021 trials are presented. 2461 trials were established, but results were obtained from 1877 (76%) reflecting various challenges.

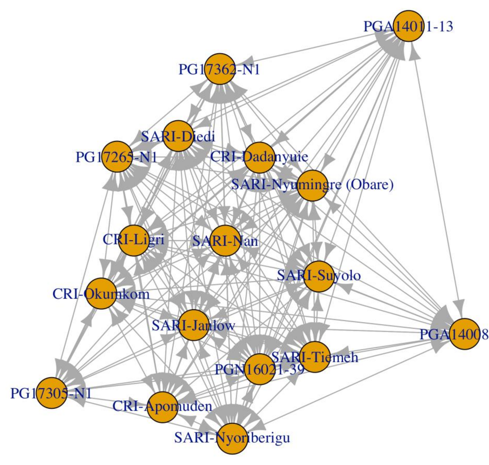

Experimental network representation of varieties tested in this trial. 2 varieties were only in south trials

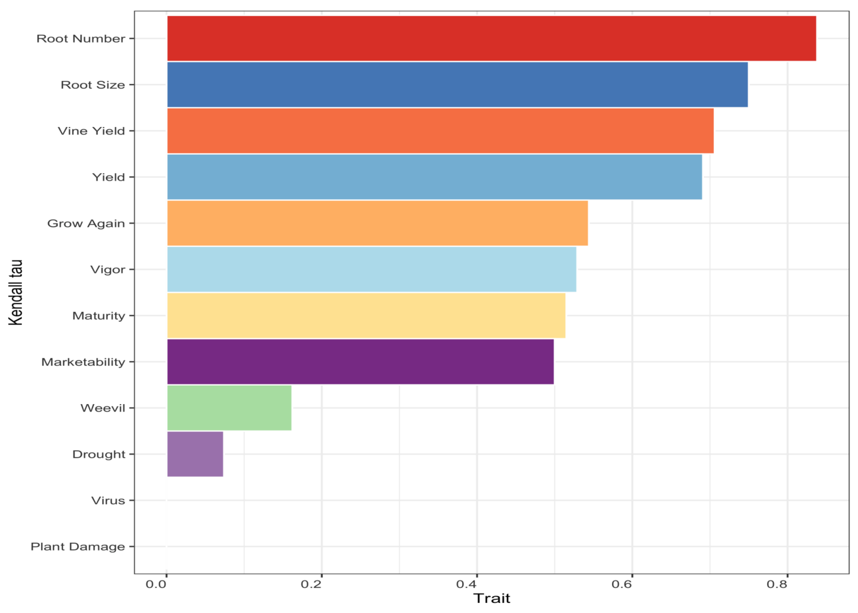

Correlation between 'Overall preference' and the other traits assessed in this trial.

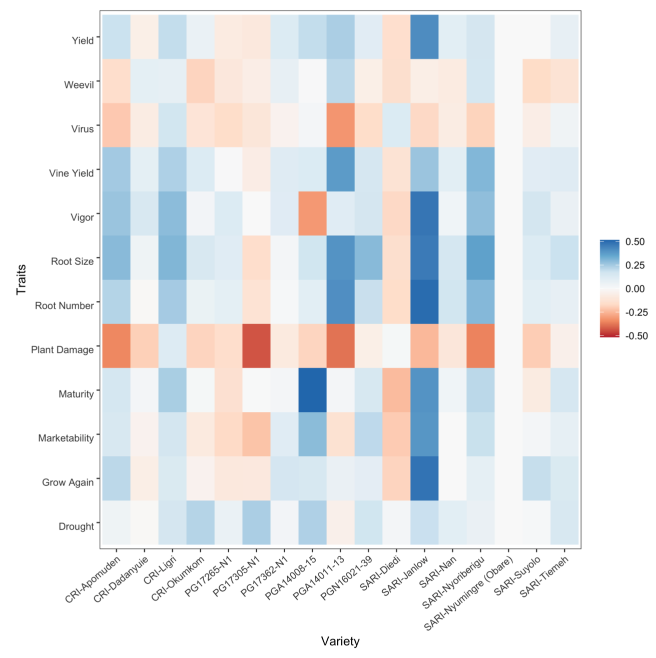

Variety performance by trait. Log-worth values where worth > 0 (blue); worth < 0 (red). SARI-Nyumingre is set as refence (log-worth arbitrarily set to zero). Overall SARI-JanLow did very well.

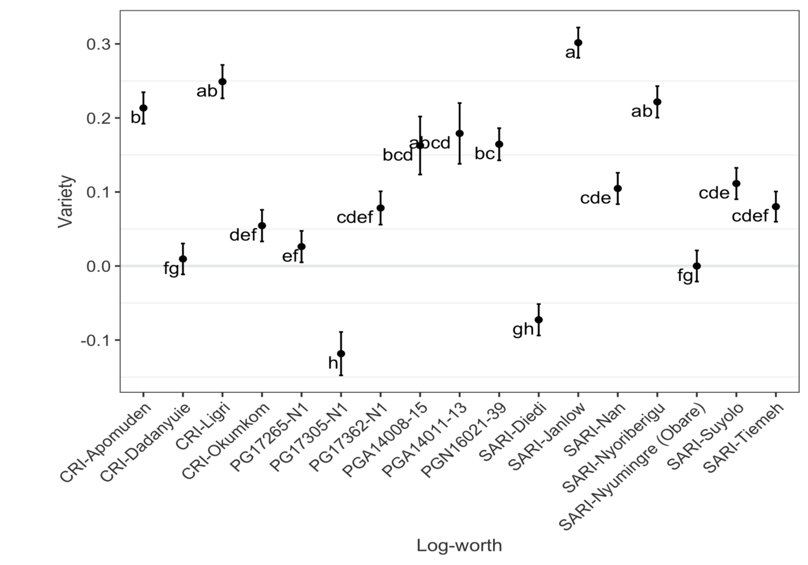

Plackett-Luce Model estimates (log-worth) of tested varieties obtained from grouped rankings combining the performance of varieties in the 13 traits assessed in this trial. The variety SARI-Nyumingre is set as reference (log-worth arbitrarily set to zero).

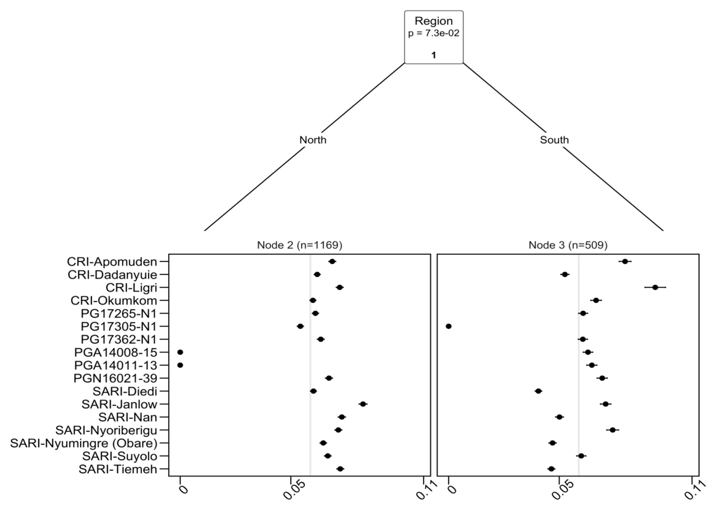

Plackett-Luce tree of tested varieties. The horizontal axis of each panel is the log-worth. Error bars show quasi-SEs. Northern and southern trials ranked differently. The significant differences among the AEZs are already well known. More in-depth analysis will reveal critical environmental factors leading to these differences.

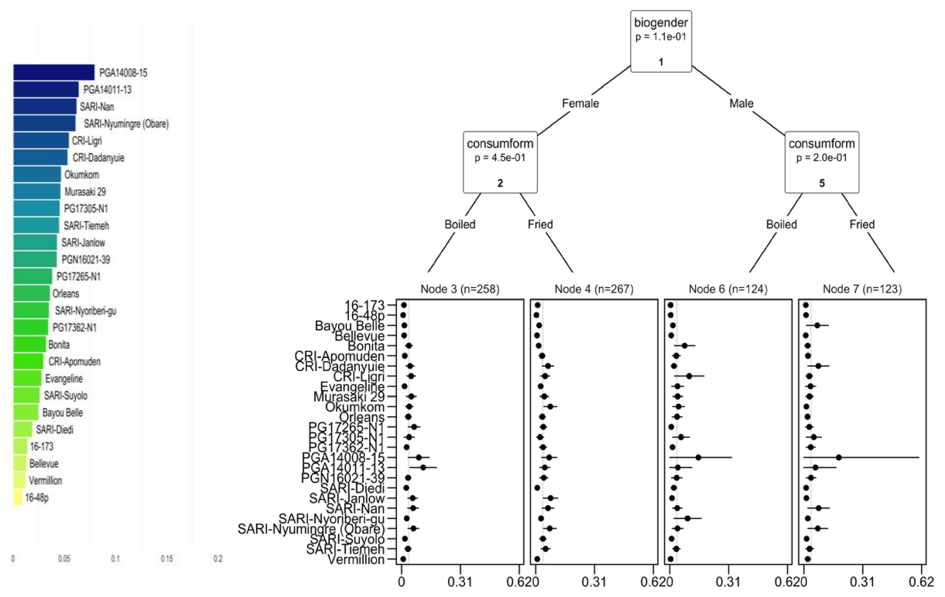

Plackett-Luce tree and overall log-worth from consumer sensory evaluation of boiled and fried sweetpotato. Men and women had distinct preferences as previously confirmed. Advanced selections were overall most preferred, boding well for variety release. Exotic OFSP introductions ranked low.

Conclusions

- Tricot was relatively easy to implement at scale, generating highly informative results.
- Department of Agriculture extension agents were the most reliable collaborators on our trials because of flexibility in their operating budgets. Schools, local NGOs and larger commercial farms using outgrowers did not provide a high success rate. Market distribution of variety packages and consumer assessment was quite successful.
- The ClimMob platform will analyze and report individual trials, but combined analysis requires more in-depth analysis using R. Further analysis will allow us to probe the phenological basis of environmental responses over trial sites.

References

Misiko, M. (2013). Dilemma in participatory selection of varieties. *Agricultural Systems* 119: 35-42.

de Sousa, K., B. Madriz, A. Muller, J. van Etten (2022) Workflow for data analysis and report with experimental data generated by crowdsourced citizen science. *Zenodo*. **v1.2**, doi:10.5281/zenodo.3976631

van Etten, J., R. Manners, J. Steinke, E. Matthus, K. de Sousa, *The tricot approach: Guide for large-scale participatory experiments* (Bioversity International; Bioversity International, Rome, Italy, 2020; https://hdl.handle.net/10568/109942).

Research was conducted under a scaling project of the CGIAR Research Program on Roots Tubers and Bananas; Department of Agriculture and other partners are greatly appreciated for their dedication to helping reach farmers. The Reputed Agriculture 4 Development Foundation sponsored some of the authors to attend the APA Conference on-line and in person.

Poster presented on APA Conference
27th June – 1st July 2022
Lilongwe, Malawi
